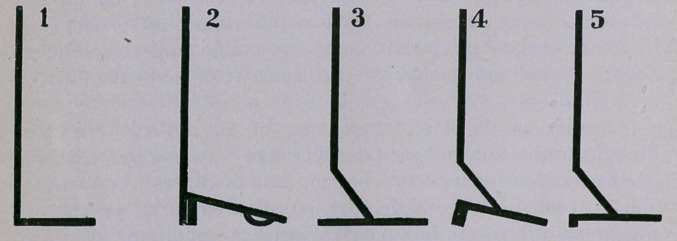# Problems in the Study of Horse-shoeing

**Published:** 1894-03

**Authors:** Williamson Bryden


### PROBLEMS IN THE STUDY OF HORSE-SHOEING.

By Williamson Bryden, V. S.

No. 1 is assumed to represent the leg, heel and foot of *Man.*

No. 2 is the same, shod with a high heel. It makes the advance of the limb easier; it also supports and relieves the strain on the tendons, etc.

No. 3 is assumed to represent the leg, ankle, pastern and foot of the *Horse.*

No. 4 is No. 3 shod with a high heel, which lowers the ankle, diminishing the space between it and the heel; it also throws more strain on the tendons, and retards the advance.

No. 5 is the same as 3 and 4, but shod with calkers all the same height; the toe corks are set back to about the second nail holes, accordingly as the ankles are straight or hang back over the heels, It therefore eases the tendons and makes the advance also easier, but it *shortens the step*, which is favorable to it, especially in overreaching, in cockling, in spring knees, etc. In open weather, when corks are *not* required on driving horses, I use a plain shoe swedged down at the toe.

My object in offering these diagrams is to call attention to the difference between the leg and foot of *man* and the leg and foot of the horse. In comparing No. 2 with No. 4, a serious blunder is made apparent. The high heel in man is quite correct, but when applied to the digit of the soliped it is illogical ; such adjustment being always a mistake, and one that has done much mischief to the horse's limbs and feet. That such a stupid blunder as a high heel and low toe, or a low heel and high toe, should become popular is really marvellous, almost as much so as the *notion* that to reduce the wall, sole, bars and frog should be regarded as almost a *criminal offence.*

## Figures and Tables

**Figure f1:**